# Genome-wide identification and expression analysis of phosphate-sensing SPX proteins in oats

**DOI:** 10.3389/fgene.2024.1469704

**Published:** 2024-11-20

**Authors:** Yinke Du, Jie Gong, Ziyi Dou, Wei Zheng, Renwei Sun, Shiqing Gao

**Affiliations:** ^1^ College of Grassland Science, Xinjiang Agricultural University, Urumqi, Xinjiang, China; ^2^ Institute of Hybrid Wheat, Beijing Academy of Agriculture and Forestry Sciences (BAAFS), Beijing, China; ^3^ Laboratory of Plant Breeding and Genetics, Department of Agricultural and Environmental Biology, The University of Tokyo, Tokyo, Japan

**Keywords:** phosphorus, salt, ABA treatments, *AsSPX* gene family, *Avena sativa* L

## Abstract

Phosphorus is indispensable to plant growth and development. Soil phosphorus deficiency poses a substantial constraint on crop yield. SPXs play pivotal roles in phosphate transport and absorption in plants. Yet, the functions of SPXs of oat *(Avena sativa* L.) under abiotic stresses remain unclear. In this study, we conducted a genome-wide analysis of 169 *SPXs* from hexaploid oat and five closely related plant species. All homologous *AsSPXs* were found to arise from duplication events and depict a strong purifying selection. Subcellular localization prediction revealed that AsSPXs were mainly located on the plasma membrane. Seventeen *cis*-acting elements, predominantly comprising light-, low temperature-, abscisic acid-, and drought-responsive elements, were dispersed in the promoter regions of *AsSPXs*. Analysis of *cis*-regulatory elements, protein-protein interaction networks, and qRT-PCR showed that *AsSPXs* are not solely involved in phosphorus starvation response but also in various stress responses. Notably, *AsSPX18-5D* (*AVESA.00001b.r3.5Dg0002895*) exerted pivotal roles in conferring resistance against low phosphorus, salt, and ABA treatments. Our study aimed to explore important stress-resistant genes in oat. Our results could provide a basis for future studies on the evolution and functions of the *AsSPX* gene family and a crucial foundation for comprehending how oat responds to environmental stresses.

## 1 Introduction

Global climate changes, such as soil salinization, drought, extreme temperatures, etc., have worsened over the years (Borrelli et al., 2017; He et al., 2023), leading to a decline in the yield and quality of crops. Thus, finding new ways to improve the yield of forage crops under adverse environmental conditions has become a primary concern worldwide. Oat (*Avena sativa* L.) is an annual herbaceous plant belonging to the Poaceae family. It exhibits remarkable adaptability, thriving in various adverse conditions, such as impoverished soil, saline-alkali, drought, and severe cold stresses. Therefore, oat has become an important genetic resource for studying plant adaptations to harsh environments (Dangi, 2020). It also serves as an ecological forage grass, playing an important role in soil salinization and desertification control, supporting the advancement of the grassland livestock industry (Dangi, 2020). Understanding the response mechanisms of plants to different stresses can help develop practical interventions to improve crop yield.

The SPX proteins, phosphorus receptors, play a crucial regulatory role in plant growth and development. An SPX protein derives its name from the initials of three proteins: Suppressor of yeast gpa 1 (SYG1), phosphate-regulated cyclin-dependent kinase inhibitor 81 (Pho81), and xenotropic and polytropic retrovirus receptor 1 (XPR1) (Wang et al., 2004). SPXs are characterized by the presence of a conserved SPX domain, which exhibits hydrophobic properties, is situated at the N-terminal, and is approximately 165 amino acids (aa) long (Chiou and Lin, 2011; Secco, et al., 2012b). The SPX protein family is divided into four distinct subfamilies: SPX proteins, which contain only the SPX domain and primarily are regulators of plant phosphate starvation (Zhang et al., 2016); SPX-EXS proteins, which contain both the SPX and EXS (deriving its name from the initials of endoplasmic reticulum retention defective 1 (ERD1), XPR1, and SYG1) domains and play an important role in phosphate acquisition, translocation, and assignment (Wang et al., 2004; Zhao et al., 2019); SPX-MFS proteins, which contain the SPX domain bound to the major facilitator superfamily (MFS) domain and are a newly discovered class of vacuolar Pi transporters in plants (Wang C. et al., 2012); and the SPX-RING proteins, which contain the SPX and really interesting new gene (RING)-type zinc finger domains and are involved in phosphate homeostasis and inorganic phosphate (Pi) reaction (Yang et al., 2017; Yue et al., 2017).

Numerous studies have shown that SPX proteins play an important role in the absorption, transport, storage, and signal transduction of Pi in plants. For instance, in *Arabidopsis thaliana*, *AtSPX1/3* (*AtSPX1/3*), *AtSPX2*, and *AtSPX4* have been shown to be strongly induced, weakly induced, and inhibited, respectively, under low-phosphorus stress conditions (Duan et al., 2008; Secco, et al., 2012a). In rice (*Oryza sativa*), *OsSPX1/2/3/5/6* are induced by phosphorus deficiency in the aerial parts of the plant and the roots. When these genes are simultaneously overexpressed with rice *phosphate starvation response 2* (*OsPHR2*), the phosphorus accumulation phenotype can be restored to wild-type levels (Lv et al., 2014; Shi et al., 2014; Wang et al., 2009; Wang et al., 2014; Zhong et al., 2018). OsSPX1 and OsSPX2 interact with OsPHR2 via their SPX domains and regulate the binding of OsPHR2 to the PHR1-binding sequence (P1BS; GNATATNC). The functional redundancy of this process is phosphorus-dependent, and this process inhibits the phosphate starvation response (Wang et al., 2014). Similarly, in wheat (*Triticum aestivum*), TaSPX1/5 have been found to interact with TaPHR3 (Sun R. et al., 2023). In addition, nitrogen signaling also plays an important role in regulating the accumulation of SPX proteins. A previous study reported that under high nitrogen conditions, nitrate transporter 1.1 (NRT1.1), the nitrate sensor in rice and Arabidopsis, binds to SPX4 and recruits NBIP1, an E3 ubiquitin ligase, to degrade it. Furthermore, high nitrogen induces *nitrate-inducible GARP-type transcriptional repressor 1 (NIGT1*), and the NIGT1 protein, in turn, inhibits the expression of SPXs and promotes phosphorus absorption, mediating nitrogen-phosphorus balance in plants (Hu and Chu, 2020; 2019; Kiba et al., 2018; Ueda et al., 2020).

In a saline environment, there can be synergy or antagonism between the salt and nutrient levels in plant roots, resulting in imbalances of several nutrients, such as phosphorus and nitrogen, affecting plant growth and development and accelerating plant aging (Ahmad et al., 2013; Wang et al., 2021). In cotton, *Cot-AD50458* encodes SPX1-like protein, and under salt stress, this gene is significantly downregulated, inhibiting phosphorus absorption and accelerating plant aging (Wang et al., 2021). Several phosphate starvation-inducing genes are expressed in senescent leaves of *A. thaliana*. For instance, *AtSPX1* overexpression accelerates leaf senescence, suppresses Pi accumulation, promotes salicylic acid (SA) production, and enhances H_2_O_2_ levels in *A. thaliana* leaves (Yan et al., 2019). Oat exhibits a strong ability to adapt to harsh environments. A previous study showed that drought-resistant oat varieties exhibited elevated abscisic acid (ABA) levels and reduced stomatal conductance, resulting in lower transpiration rates and greater drought resistance (Canales et al., 2021). Another study showed that the overexpression of salt-induced CBF3 in oats can delay leaf aging, reduce the loss of yield, and enhance tolerance to abiotic stresses (Oraby and Ahmad, 2012). OsSPX1 is an important link between the signaling pathways related to low temperature and Pi starvation stresses. *OsSPX1* overexpression has been shown to enhance cold tolerance and reduce the total phosphorus content in the leaves of tobacco and *A. thaliana* (Zhao et al., 2009). Conversely, *OsSPX1* downregulation increases the sensitivity of rice seedlings to both cold and oxidative stresses (Wang et al., 2013).

Members of the *SPXs* have been found across several plant species, including *A. thaliana* (Duan et al., 2008), rice (Wang et al., 2009), wheat (Kumar et al., 2019), corn (Xiao et al., 2021), tomato (Li et al., 2021), *Brassica napus* (Du H et al., 2017), giant duckweed (Yang et al., 2022), moso bamboo, and tea oil camellia (Chen et al., 2023; Luo et al., 2023). As core regulatory proteins in phosphorus signaling pathways, SPX proteins have been extensively studied in nitrogen-phosphorus signaling (Hu et al., 2019). The release of high-quality reference genomes for heterohexaploid oat variety “Sanfensan” (*A. sativa* ssp. nuda, AACCDD, 2n = 6x = 42) and oat “sang” (*A. sativa* L., AACCDD, 2n = 6x = 42), and their diploid (*A. longiglumis*, AA, 2n = 14) and tetraploid ancestors (*A. insularis*, CCDD, 2n = 4x = 28) has accelerated the process of genome-assisted oat breeding (Kamal et al., 2022). However, to the best of our knowledge, genome-wide identification and characterization of members of the SPX gene family in oats have not yet been reported. This study aimed to perform a comprehensive identification and functional analysis of the SPX gene family in oats.

## 2 Materials and methods

### 2.1 *Identification of* SPX *genes*

The EnsemblePlants database (http://plants.ensembl.org/index.html) was accessed to obtain the genomic data of a dicot (Arabidopsis) and several monocots (oats, barley, rice, wheat, and maize). Firstly, we conducted BLASTP of 20 AtSPXs and 15 OsSPXs. The Hidden Markov Model (HMM) SPX domain (PF03105) in the Pfam database (http://pfam.xfam.org/) were used to search for *AsSPXs* genes. Then, all the obtained sequences were further verified using the Pfam and SMART (http://smart.embl-heidelberg.de) to determine if they contained SPX domain with the threshold of E < 1e-5. All the gene loci were numbered and mapped onto their corresponding chromosomes. For the polyploid oats, the gene loci were numbered based on the order of A/C/D subgroups.

### 2.2 Physiochemical analysis and subcellular localization prediction

The ProtParam (http://web.expasy.org/protparam/) program of Expert Protein Analysis System (ExPASy) was used to predict the molecular weights (MWs) and isoelectric points (PIs) of all AsSPX proteins (Garg et al., 2016). The subcellular localization prediction of the oat SPX proteins was predicted using the Bologna Unified Subcellular Component Annotator (BUSCA; http://busca.biocomp.unibo.it) (Savojardo et al., 2018).

### 2.3 Evolutionary analysis

To determine the evolutionary relationship among the SPX proteins, they were assessed using the Molecular Evolutionary Genetics Analysis 7 (MEGA7) software to construct phylogenetic trees using the neighbor-joining method with 1,000 bootstrap replications (Kumar et al., 2018). Multiple Collinearity Scan toolkit X (MCScan X) was used for collinearity analysis among wheat, Arabidopsis, rice, and oats to identify gene duplication events (Wang Y. et al., 2012). The syntenic relationships were determined using TBtools. The Ka/Ks ratios of collinear gene pairs were calculated using the coding sequences (CDS) and protein sequences. The divergence time was calculated using the following formula: T = Ks/2λ × 10^–6^ Mya (λ = 6.5 × 10^−9^) (Gaut et al., 1996).

### 2.4 Protein-protein interaction (PPI) network analysis

The conserved motifs and domains in the SPX proteins were identified using the Multiple Em for Motif Elicitation (MEME) (https://meme-suite.org/) and conserved domain (CD) searches (https://www.ncbi.nlm.nih.gov/). The potential cis-acting elements were analyzed via the PlantCARE (https://bioinformatics.psb.ugent.be/web--tools/plantcare/html/) database using the 2 kb region upstream of the start codon in all the AsSPXs (Lescot et al., 2002). Finally, the integrated results were visualized using the Gene Structure View function of TBtools. The PPI network of 42 AsSPX protein sequences was constructed using the STRING database. Due to the lack of a protein interaction database of oats, we analyzed the homologous SPX proteins in wheat to predict the interactions among AsSPXs. The Cytoscape software was used to visualize the PPI networks.

### 2.5 Plant materials and growth conditions

Hexaploid oat plants were used in this study, courtesy of Professor Zhao Guiqin from Gansu Agricultural University. The oat seeds were disinfected using 75% alcohol for 5 min and germinated for 5 days in the growth chamber. The germination conditions included 50% relative humidity and 14-h day/10-h night cycles with temperatures of 24°C/18°C, respectively. The germinated seeds were then cultured in a modified Hogland solution (NS10205-100 mL, Coolaber) for 15 days. Finally, the seedlings were transferred to the following six Hoagland solutions for 24 h for Pi, salt, drought, ABA, cold and heat stress: 0 mol/L Pi (NSP1020-P-500L, Coolaber), 0.2 mol/L sodium chloride (NaCl, Hushi), 20% polyethylene glycol 6,000 (PEG_6000_, Solarbio), 30 μmol/L abscisic acid (ABA, Sigam), 4°C and 40°C. Hoagland’s solution without any treatment was used as a control (CK). The shoots/leaves and roots of the different groups of seedlings were collected at 0, 1, 3, 6, 9, 12, and 24 h, and immediately frozen in liquid nitrogen and stored in a −80°C freezer.

### 2.6 RNA isolation, cDNA preparation, and quantitative real-time polymerase chain reaction (qPCR)

Total RNA was extracted from plant extracts using a FastPure^®^ Universal Plant Total RNA Isolation Kit (Vazyme, Nanjing, China) as per the manufacturer’s instructions. First-strand cDNA synthesis was performed using a HiScript^®^ III 1st Strand cDNA Synthesis Kit (+gDNA wiper) with gDNA Eraser (Vazyme, Nanjing, China). qRT-PCR was conducted using a CFX96 Touch™ Real-Time PCR Detection System (Bio-Rad Laboratories, Hercules, CA, United States) with HiScript^®^ III All-in-one RT SuperMix Perfect for qRT-PCR (Vazyme, Nanjing, China). Primers were designed using the Primer3plus online tools (http://www.primer3plus.com/) (Supplementary Table S1). Expression levels of target genes were normalized against oat 18S RNA. The relative expression levels were calculated using the 2^−ΔΔCT^ method (Livak and Schmittgen, 2001).

## 3 Results

### 3.1 Identification and analysis of AsSPXs

**FIGURE 1:**
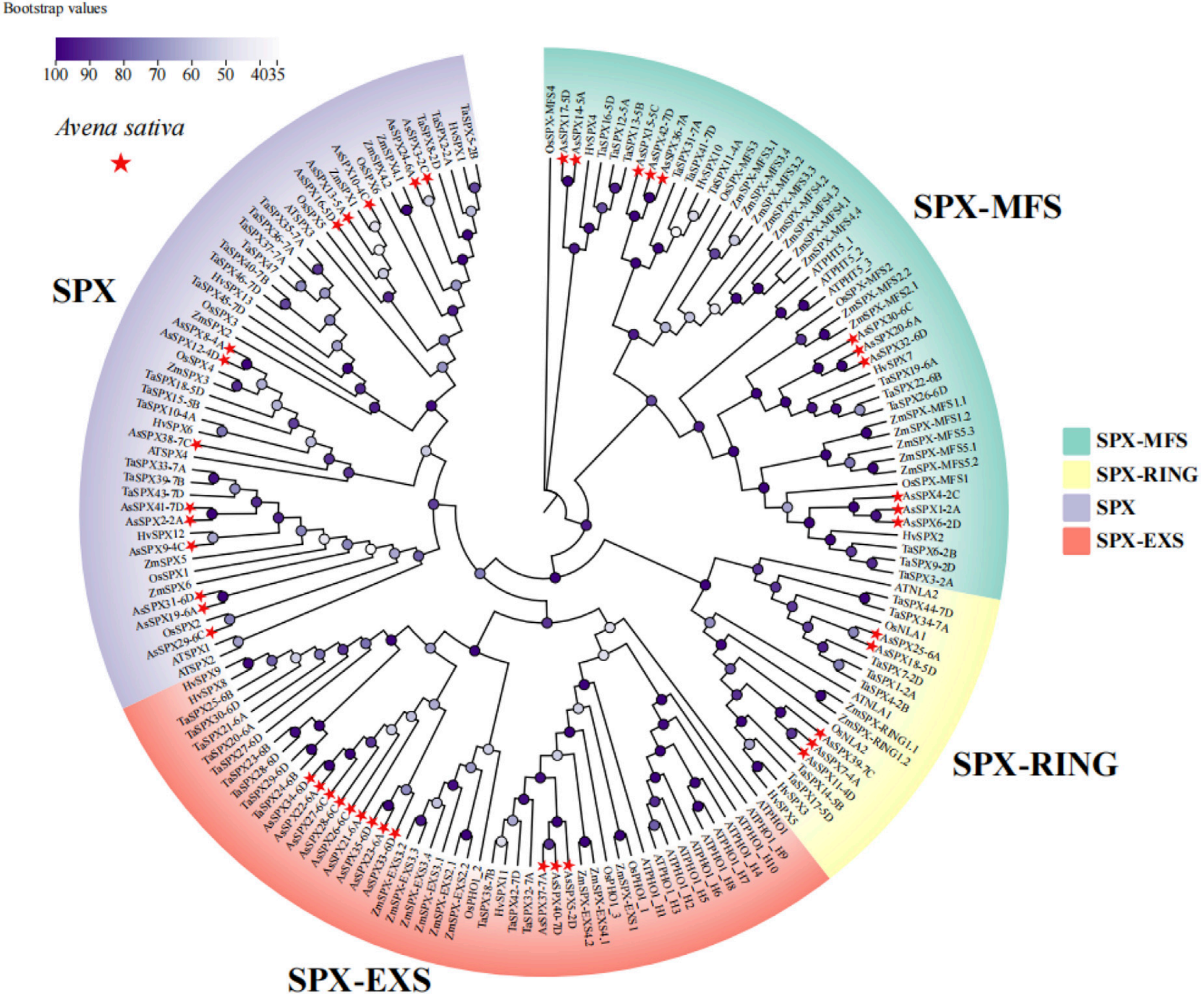
Phylogeny of neighborhood junctions of 169 SPXs from six plant species, *Avena sativa, Arabidopsis thaliana, Hordeum vulgare, Oryza sativa, Triticum aestivum, Zea mays* (n = 42, 20, 13, 15, 46, and 33 SPXs, respectively). AsSPXs were divided into four subfamilies based on their domains: SPX, SPX-EXS, SPX-MFS, and SPX-RING (comprising 14, 12, 11, and five AsSPXs, respectively). *AsSPXs* are represented by a red triangle.

**FIGURE 2:**
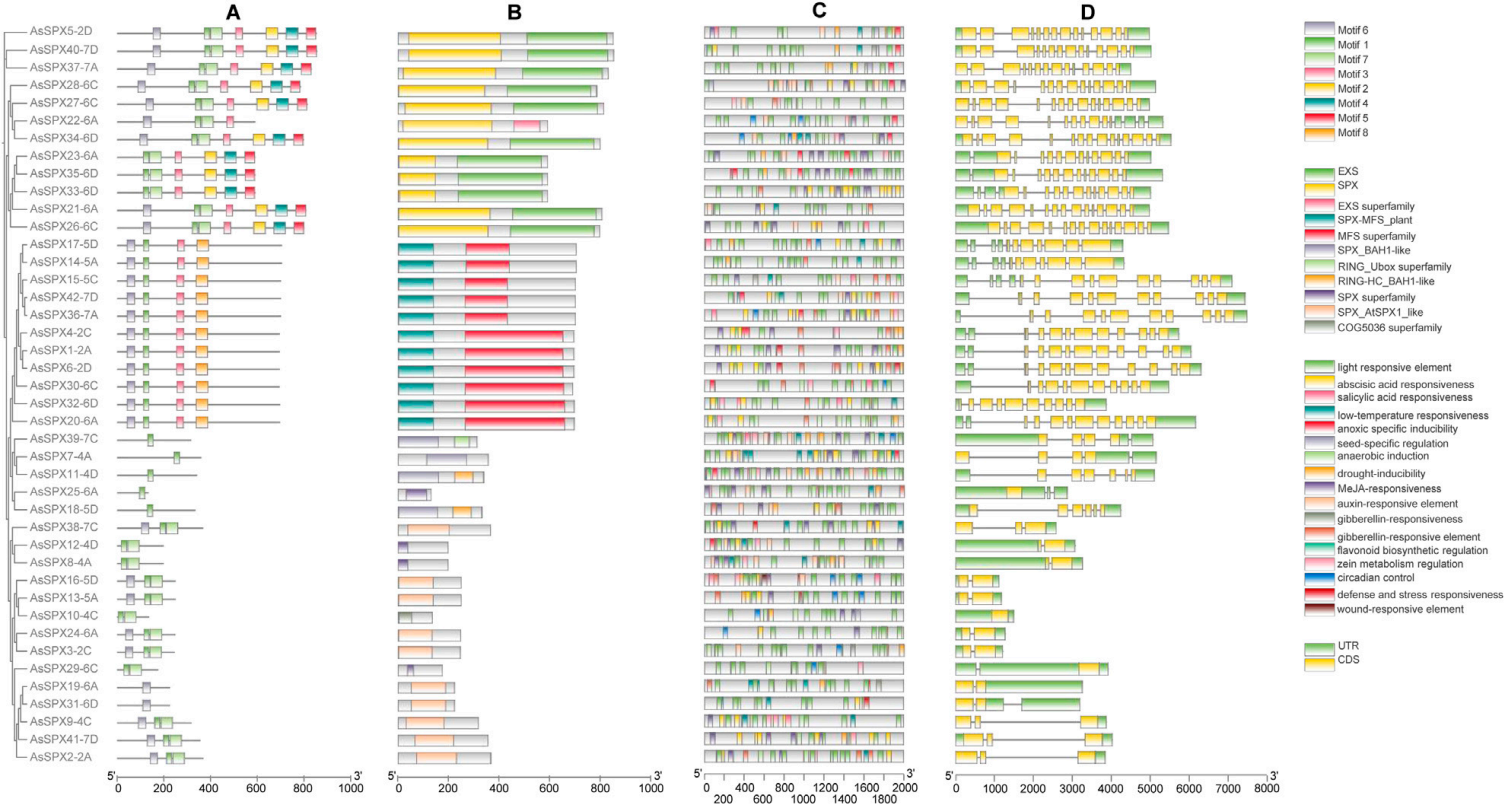
Analysis of phylogeny, motifs, cis-elements in promoters, and gene structures of all 42 AsSPXs. **(A)** Protein motifs, **(B)** Protein domains, **(C)** Cis-elements in the promoters, **(D)** Gene structures.

We identified a total of 42 AsSPXs via conserved domain analysis and sequence alignment. According to the chromosome where each gene is located on the chromosome, numbered AsSPX1-2A ∼ AsSPX42-7D (Figures 1, 2; Supplementary Table S2). The lengths and molecular weights (MWs) of the identified AsSPXs were in the range of 131–854 aa and 14,709.15∼97,379.45, with the longest and bulkiest variant being AsSPX40-7D, and the shortest and least bulky being AsSPX25-6A. The isoelectric points (PIs) of the identified AsSPXs ranged from 5.27 to 9.27, with an average PI of 7.52, with acidic, basic, and neutral aa accounting for 42.86%, 40.48%, and 16.67%, respectively. The substantial variations in the lengths and properties of AsSPXs proteins indicated that their acidic, alkalinity properties and non conservatism. Subcellular localization prediction revealed that most AsSPXs are mainly located on the plasma membrane, followed by the cytoplasm and nucleus, and a few were located in the mitochondria. These findings suggested that AsSPX family members exhibit multiple biological functions and redundancy mechanisms.

### 3.2 Phylogenetic analysis of the SPXs from oat and other species

To elucidate the evolutionary relationships among the SPXs across different plant species, we assessed a total of 169 SPXs from *A. sativa*, *A. thaliana*, barley (*Hordeum vulgare*), rice, wheat, and maize (*Zea mays*) (n = 42 AsSPXs, 20 AtSPXs, 13 HvSPXs, 15 OsSPXs, 46 TaSPXs, and 33 ZmSPXs, respectively). Using the neighbor-joining method, we constructed a phylogenetic tree of all SPXs (Figure 1). AsSPXs were divided into four groups based on their domain sequences, SPX, SPX-EXS, SPX-MFS, and SPX-RING subfamilies, comprising 14, 12, 11, and five members, respectively (Figures 1, 2). Similar to oats, most TaSPXs, HvSPXs, and OsSPXs belonged to the SPX group, followed by SPX-EXS, SPX-MFS, and SPX-RING subfamilies. Therefore, the SPXs of these plants harbored conserved SPX domains, thus functioning as regulators of plant phosphate starvation. In contrast, fifteen, nine, seven, and two ZmSPXs were categorized into the SPX-MFS, SPX, SPX-EXS, and SPX-RING subfamilies, respectively. Furthermore, most AtSPXs belonged to the SPX-EXS group (55%), and the fewest belonged to the SPX-RING subfamily (10%). Thus, for all species, the SPX regions were relatively scattered, while the SPX-EXS and SPX-MFS regions were relatively clustered. Since oat, wheat, and barley belong to the Poaceae family, oat is evolutionarily closer to wheat and barley, indicating that their SPX gene families are highly conserved and can be used to assess the evolution and biological functions of the *SPX* genes.

### 3.3 *Gene structure and conserved motif analysis of* AsSPXs

To identify commonly occurring motifs in SPXs, we predicted the composition of the 42 AsSPXs using the MEME tool and identified eight distinct motifs (Figure 2; Supplementary Table S3). Notably, motif 1 was present in all SPXs except for AsSPX19-6A and AsSPX31-6D, implying its potential role in their shared functionality. The SPX-RING group only possessed motif 1. The SPX-MFS subfamily contained motifs 1, 3, 6, and 8; however, motif 8 was solely found in the SPX-MFS subfamily. Motifs 2 to 5 were exclusively present in the SPX-EXS subfamily, with the group members harboring one to seven motifs. These findings indicated that proteins within the same subfamily exhibited similar distribution patterns of motifs. In addition, specific regulatory motifs in the members of each group might contribute to their distinct functions observed across the subfamilies. To comprehensively understand the evolutionary relationships and functional diversity among *AsSPXs*, we analyzed the intron-exon boundaries within their sequences. We observed that all the genes harbored introns except for the gene encoding *AsSPX10-4C*. Additionally, 10 genes, such as *AsSPX37-7A*, *AsSPX27-6C*, and *AsSPX22-6A*, lack 5′ introns. This absence is hypothesized to be the result of selective pressures during evolution, where efficient expression for specific functions and increased protein production may have led to the loss of 5′ introns. Furthermore, the SPX-EXS, SPX-MFS, SPX-RING, and SPX group members contained 12–14, 9–13, 2–6, and two introns, respectively. The same subfamily performs similar functions and therefore has similar structures, such as the AsSPX2-2A and AsSPX9-4C genes, which belong to the same SPX subfamily. These results indicated the conservation of gene structure within each SPX subfamily along with the presence of complex structures, implying other potential functionalities associated with the *SPX* genes.

### 3.4 *Analysis of the cis-acting elements of* AsSPXs

*Cis*-regulatory elements play a crucial role in gene expression regulation. Analysis of the sequences 2000 bp upstream of each AsSPX gene revealed a total of 17 predicted cis-acting elements, providing insights into the regulatory elements of the AsSPX gene family. (Figure 2; Supplementary Table S4). They were categorized into three main groups based on whether they were related to hormone response (n = 5; responsive to ABA, SA, methyl jasmonate (MeJA), auxin, and gibberellin), abiotic stress response (n = 6; responsive to low-temperature, anoxic conditions, anaerobic, drought, defense reactions, and wound), and plant development (n = 4; responsive to seed development, flavonoid biosynthesis, zein metabolism, and circadian control). Similar to the members of the *TaPHR* (Kumar et al., 2019), *ZmSPX* (Xiao et al., 2021), and *SlPEBP* (*PEBP* gene family of tomato (*Solanum lycopersicon*)) (Sun Y. et al., 2023) gene families, light-responsive elements were significantly enriched in the promoters of all *AsSPXs* suggesting their potential involvement in the same signaling pathway. Furthermore, low-temperature-responsive elements were identified in 22 genes, suggesting the crucial role of SPXs as a connecting link between cryogenic stress and Pi starvation signaling pathways (Wang et al., 2013; Zhao et al., 2009). ABA-, MeJA-, and drought-responsive elements were present in 39, 33, and 29 genes, respectively. Though several *SPXs* are known to be induced by Pi starvation, the presence of P1BS (a conserved Pi stress-responsive element) in *SPXs* of oat or apple (*Malus pumila*) has not yet been reported (Kural et al., 2024).

### 3.5 Collinearity analysis of the gene duplication events in AsSPXs

**FIGURE 3:**
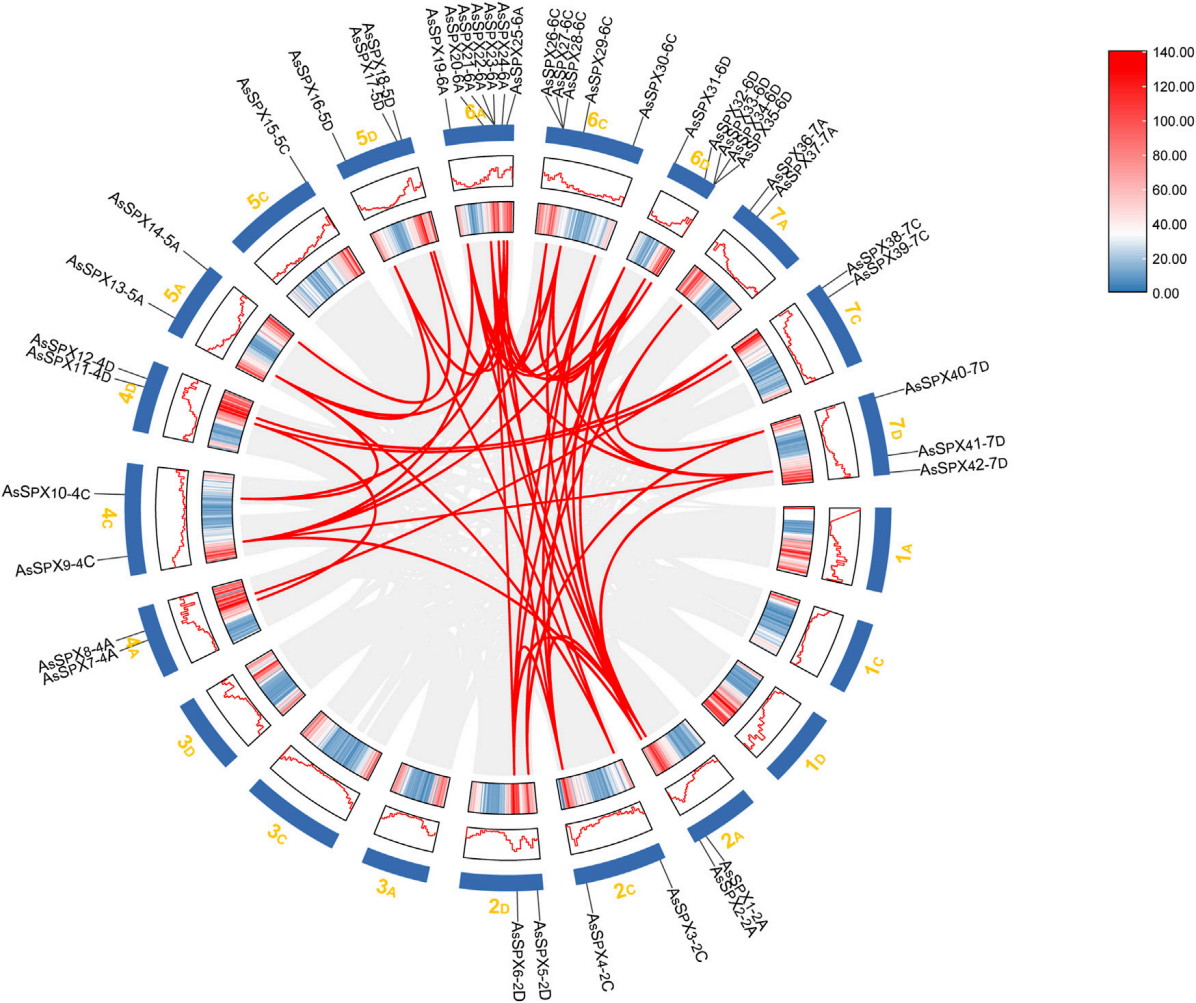
Collinearity analysis of the *SPX* gene family in oat. The chromosomal distribution of 42 *AsSPXs* is depicted. Chromosome size and gene density plots are shown in the Circos plot, represented by different shades as plots and heat maps. The collinear relationships among the genes were scanned with MCScanX and connected with red lines.

Genomic collinearity segments within the oat genome were comprehensively analyzed to infer gene replication events during the evolution of the AsSPX protein family. All *AsSPXs* are mainly derived from genome-wide replication (whole genome duplication; WGD) or fragment replication, followed by tandem duplication (Supplementary Table S5). The 42 identified *AsSPXs* were distributed across 14 oat chromosomes, with each chromosome harboring a varying number of *AsSPXs* (Figure 3). Among them, the sixth homologous group harbored the most genes (n = 17). The first and third homologous groups exhibited an uneven gene distribution; however, the second, fourth, fifth, and seventh homologous groups exhibited a relatively stable distribution with 6-7 genes in each group. We detected a total of 35 collinear gene pairs in the oat genome. Furthermore, *AsSPXs* located on different oat chromosomes exhibited collinearity. This finding suggested that most *AsSPXs* might be alleles or paralogous genes on homologous chromosomes and have undergone genetic expansion during their evolution. For example, AsSPX1-2A exhibited collinear relationships with AsSPX4-2C, AsSPX6-2D, AsSPX20-6A, AsSPX30-6C, and AsSPX32-6D. Additionally, we observed collinearity between 4C (AsSPX9-4C) and 2A (AsSPX2-2A). These findings suggested that AsSPXs might have evolved from gene replication processes during evolution, with the encoded proteins influenced by varying degrees of truncated duplication events.

**FIGURE 4:**
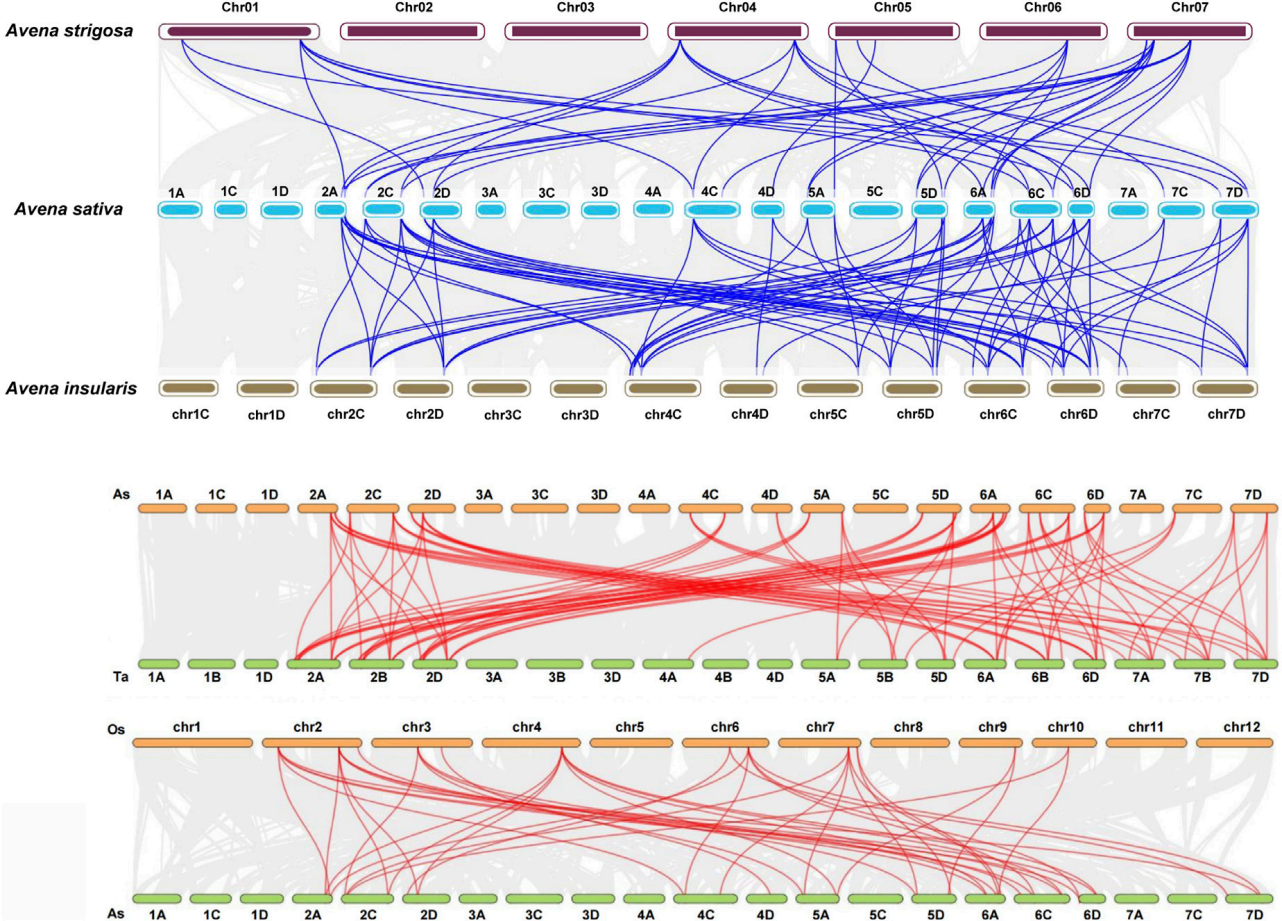
Collinearity analysis between oat and other plant species. **(A)** There were 41 and 79 pairs of homologous genes between hexaploid oat and *Avena strigosa* and hexaploid oat and *Avena insularis*, respectively. **(B)** There were 93 and 44 pairs of homologous genes between hexaploid oat and wheat and hexaploid oat and rice, respectively.

The inter-specific collinearity analysis revealed the presence of 41 and 79 pairs of homologous genes between hexaploid oat and diploid *A. strigosa* and hexaploid oat and tetraploid *A. insularis*, respectively. If a gene in hexaploid oats lacks colinearity with its diploid counterpart, it similarly will not be collinear with the corresponding gene in tetraploid oats (Figure 4A; Supplementary Table S6). There are 93 homologous gene pairs between oat and wheat (Figure 4B). More specifically, orthologous gene pairs were found between the sixth and second homologous groups in oat and the second, sixth, and seventh homologous groups in wheat. Additionally, 15, nine, and eight orthologous gene pairs were detected between the fifth, seventh, and fourth homologous groups in oat and wheat. Notably, no collinearity was observed between the first and third homologous groups in oat and wheat. Furthermore, 44 homologous gene pairs were identified between oats and rice. Similar to the previous studies, we found no collinear relationships between *AtSPXs* and *TaSPXs* (Supplementary Figure S1) (Sun R. et al., 2023). Oat is are monocots, while *A. thaliana* is dicots, and they differ significantly in morphology, structure, and developmental mode, which may be the reason for the lack of collinearity between them. To further evaluate selective pressure on genes associated with these duplication events, the Ka/Ks ratio for homologous *SPX* pairs was calculated (Supplementary Table S7). The divergence time for *AsSPX* gene pairs was estimated at ∼35.58 million years ago (MYA). Additionally, all gene pairs had a Ka/Ks < 1, with an average value of 0.25, suggesting strong purification selection upon AsSPX gene pairs.

### 3.6 Protein-protein interaction (PPI) networks of AsSPXs

**FIGURE 5:**
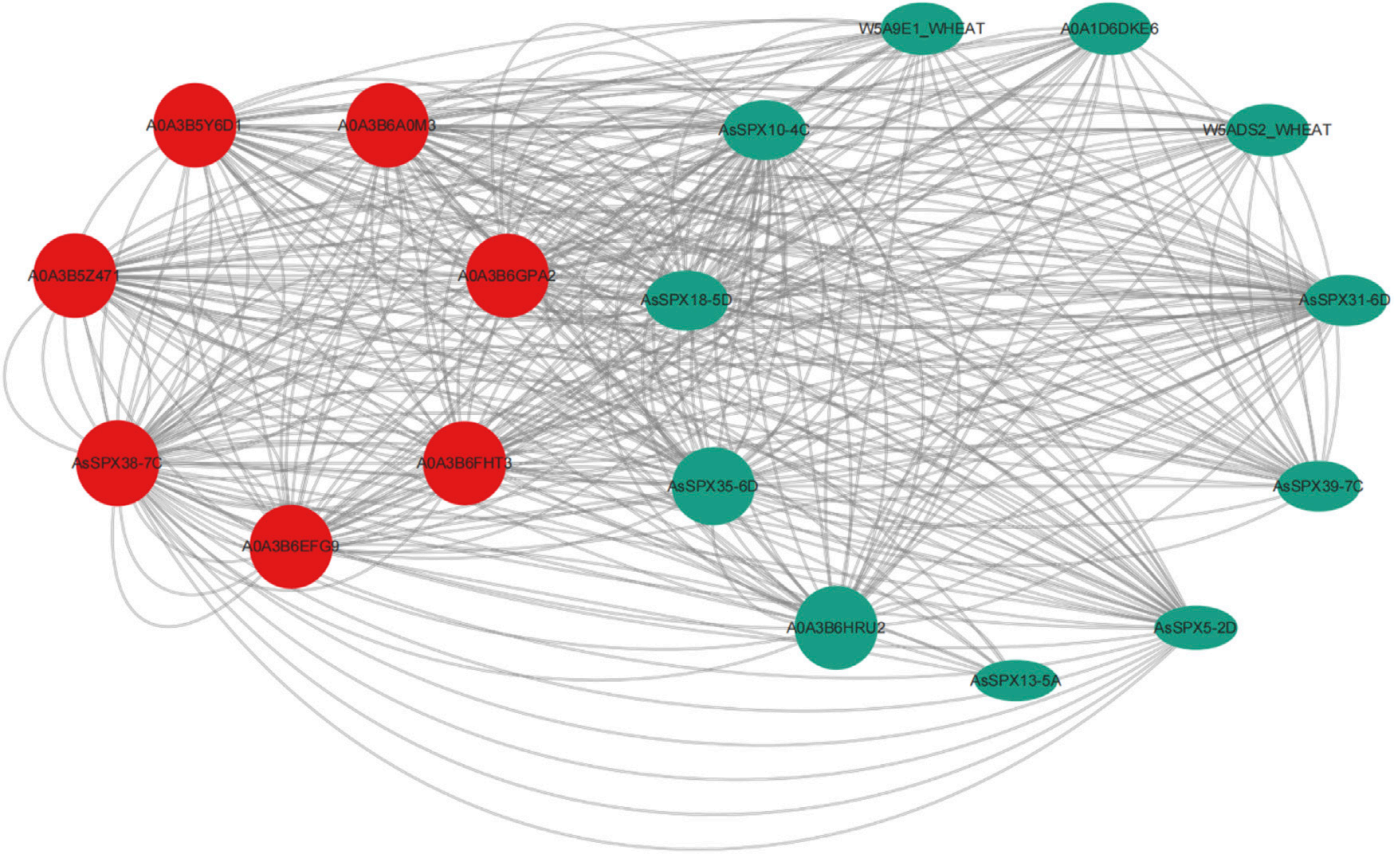
Predicted protein-protein interaction network of AsSPXs. AsSPX5-2D, AsSPX10-4C, AsSPX13-5A, AsSPX18-5D, AsSPX31-6D, AsSPX35-6D, and AsSPX39-7C reference proteins were named based on BLAST results.

Previous studies have demonstrated that *SPX4 degradation E3 ligase 1* (*SDEL1*) and *SDEL2* act as ring-fingered ubiquitin E3 ligases that regulate *OsSPX4* degradation for phosphate homeostasis and signal transduction (Ruan et al., 2019). Protein ubiquitination has also been reported within the PPI network of ZmSPX (Xiao et al., 2021). Furthermore, *AtSPX3* regulates the initiation of embryonic stem apical meristem (SAM) development by interacting with AtYABBY3, suggesting that SPX3 is involved in cell cycle regulation (Stahle et al., 2009). *OsSPX1* overexpression can enhance plant cold tolerance (Wang et al., 2013; Zhao et al., 2009). In the present study, PPI networks were used to characterize the cellular function of AsSPX. In our PPI network, seven core interacting proteins were identified: AsSPX5-2D, AsSPX10-4C, AsSPX13-5A, AsSPX18-5D, AsSPX31-6D, AsSPX35-6D, and AsSPX39-7C (Figure 5). These proteins are primarily involved in crucial biological processes, including the regulation of the phosphorus signaling pathway, protein ubiquitination, histone H3-K4 trimethylation and nucleic acid binding, response to external stimuli such as low-temperature reactions, energy and substance metabolisms such as benzoic acid and salicylic acid metabolism, and cell cycle regulation. These findings indicated that, in addition to phosphorus starvation response, these seven AsSPXs are also engaged in various stress responses.

### 3.7 Structure prediction of AsSPXs

**FIGURE 6:**
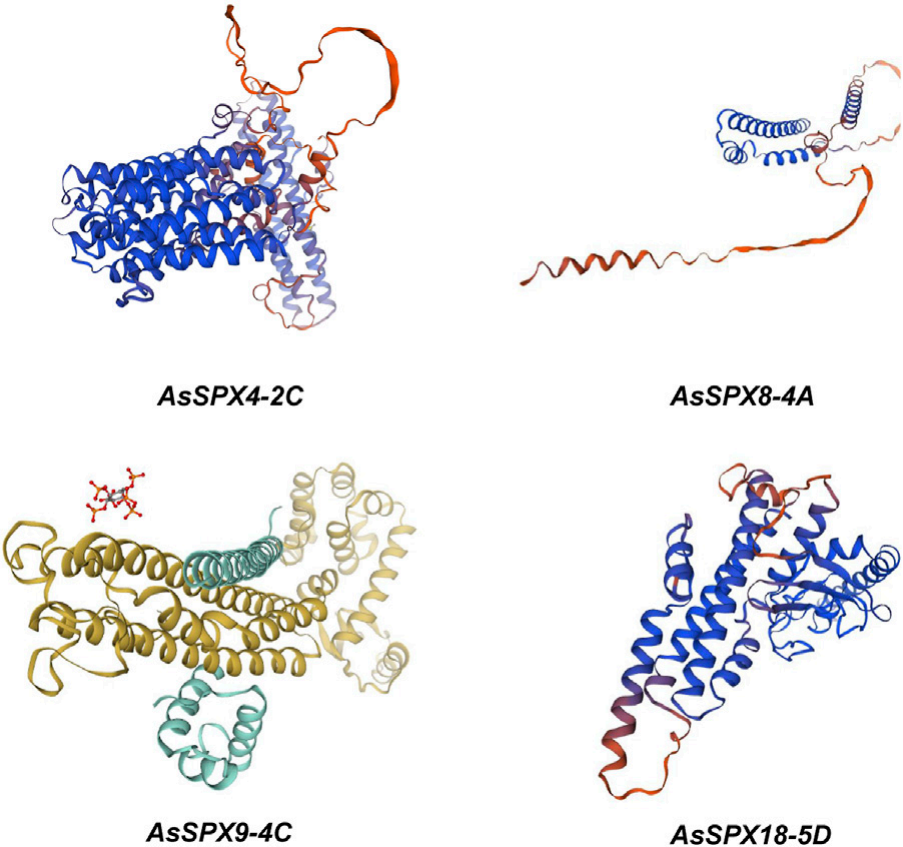
Homology modeling of phosphate starvation response (PHR) proteins encoded by four oat genes, AsSPX4-2C, AsSPX8-4A, AsSPX9-4C, and AsSPX18-5D. The program is generated through ExPaSy (https://swissmodel.expasy.org/interactive).

Through bioinformatics analysis and protein structure modeling software (Figure 6), we observed that the AsSPXs typically included a series of alpha helices and beta folds, which might play essential roles in protein function. The amphiphilic characteristics of alpha-helices and the diverse structures of beta-sheets endow proteins with a rich array of forms, enabling these four genes to adapt to a variety of environmental stresses (Sun et al., 2004). Concurrently, the complexity of alpha-helices and beta-sheets within these genes correlates with the number of exons they possess (Figure 2). A detailed analysis of the alpha helices revealed specific folding patterns that are potentially associated with the phosphate-sensing ability and signal transduction of AsSPXs. Furthermore, we performed a comparative analysis with homologous *SPXs* in wheat (Kumar et al., 2019), revealing a certain degree of similarity between the sequences of *TaSPXs* and *AsSPXs*. These findings indicated the potential involvement of TaSPXs in phosphate metabolism and signal transduction pathways. These findings are crucial to further elucidating the mechanism of action of SPXs and their role in crop growth and development.

### 3.8 *Expression of* AsSPXs *under abiotic stresses*

Oat demonstrates a strong ability to adapt to challenging environments. As a gene encoding Pi-responsive protein, the *SPX* gene plays a crucial role in regulating Pi stress responses. Previously, *OsSPX1* has been shown to be involved in low-temperature stress and phosphate deficiency, and *OsSPX1* overexpression enhances plant cold tolerance (Wang et al., 2013; Zhao et al., 2009). In *Arabidopsis*, *AtSPX1* acts as a key regulator of the pathways related to leaf senescence, phosphate starvation, and salicylic acid (SA) signaling (Yan et al., 2019). Through homologous evolution analysis, we identified functionally characterized counterparts of *SPX* genes and examined their response abilities under stress conditions, uncovering potential stress-resistance-related functional genes.

In the present study, we used qRT-PCR to analyze the expression of four *AsSPXs* under six distinct stress conditions. Compared with the control group (CK), *AsSPX4-2C* was significantly downregulated in the leaves under salt, drought, cold, and ABA stresses. However, it was induced under heat stress. In the root tissue, *AsSPX4-2C* was downregulated under drought and heat stresses. At 6 h, it was significantly suppressed in the root under salt and cold stresses.

In leaves and roots, *AsSPX8-4A* was substantially induced under salt and ABA stresses but was repressed under drought and heat stresses. Furthermore, under cold stress, the gene was generally upregulated except for the decreases at 3 h in the leaves and at 3 and 6 h in the roots.

**FIGURE 7:**
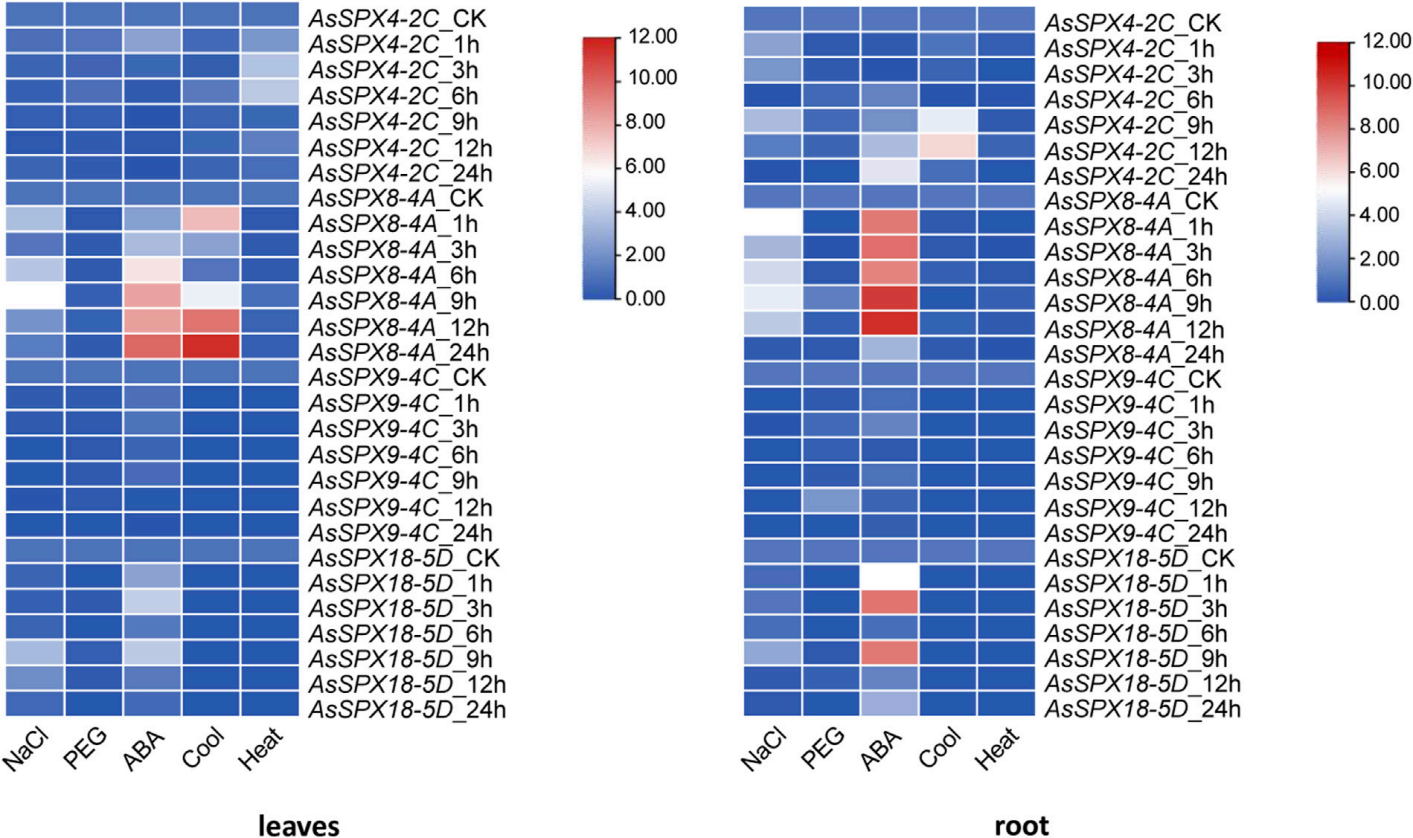
Expression heat map derived from the leaves and roots of oat plants subjected to five types of stresses. The expressions of four *AsSPXs* were analyzed after treatment with 0.2 mol/L NaCl, 20% polyethylene glycol 600 (PEG_6000_), 30 μmol/L abscisic acid (ABA), 4°C, and 40°C. Shoots/leaves and roots of the plants were collected at 0, 1, 3, 6, 9, 12, and 24 h post-treatment. In the heat map, the intensity of the red color positively correlated with the gene expression.

*AsSPX9-4C* expression was generally low, but this gene was significantly upregulated in the root tissue under drought stress at 12 h. *AsSPX18-5D* was considerably upregulated in the leaves and roots at 1, 3, and 9 h under ABA stress and mildly upregulated at 9 h under salt stress. Under heat stress, *AsSPX4-2C* was upregulated in the leaf tissue, while the other three genes were markedly downregulated in both leaf and root tissues. *AsSPX9-4C* and *AsSPX18-5D* were also downregulated under cold stress (Figure 7).

In the leaf tissues subjected to Pi stress, *AsSPX4-2C* was upregulated, with the expression increasing with time. In addition, *AsSPX8-4A* was strongly induced, especially at 12 h, while *AsSPX9-4C* and *AsSPX18-5D* were inhibited. Notably, *AsSPX9-4C* was also significantly induced at 12 h, suggesting potential functional similarities between *AsSPX9-4C* and *AsSPX8-4A*. Furthermore, since both these genes are located on homologous chromosomes, they might be involved in similar pathways.

**FIGURE 8:**
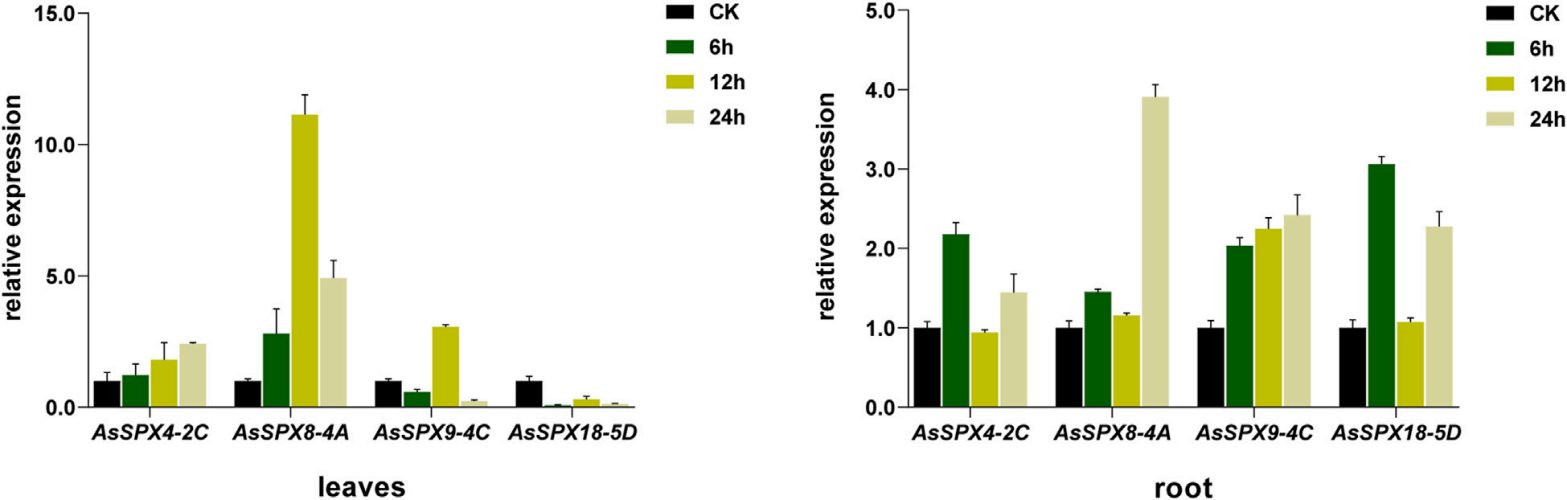
Quantitative real-time PCR of four AsSPXs isolated from plants subjected to inorganic phosphate (Pi) stress. The shoots/leaves and roots of the plants were collected at 0, 6, 12, and 24 h post-treatment. Different colors represent different sampling times.

In the root tissues subjected to Pi stress, the expressions of all four genes were generally high. Notably, *AsSPX9-4C* expression levels positively correlated with time. The remaining three genes were significantly upregulated at both 6 and 24 h, but their expressions reduced to normal levels at 12 h. This observation suggested the potential involvement of these three genes in similar pathways within the roots (Figure 8). Overall, these findings indicated that SPX plays a crucial role in response to various stresses, including Pi, drought, salt, heat, and cold stresses.

## 4 Discussion

### 4.1 Comparison of the characteristics of the SPX genes from oats and other species

As a high-quality grain and feed crop, oat exhibits resistance to extreme environments, such as salinity, drought, and cold stresses. In the past few decades, effective stress-resistance genes have been continuously excavated from different germplasm resources. Oat is also a high-quality forage and crop germplasm resource, playing an important role in improving the environment (Dangi, 2020; Paudel et al., 2021; Varma et al., 2016). In Northwest China, stresses such as drought, extreme heat, and low phosphorus severely impact plant growth and development (Borrelli et al., 2017; He et al., 2023). Phosphorus promotes seed germination and root growth, regulates nutrient uptake by plants (Ueda et al., 2020), and enhances their ability to cope with environmental stresses (Wang et al., 2013; Zhao et al., 2009). Under phosphorus-deficient conditions, plant growth is slow, root growth is stunted, flowering and fruiting are delayed, and seed setting rate is low. Molecular and genomic studies have shown that many *SPX* gene family members are involved in Pi signaling and homeostasis (Lv et al., 2014; Shi et al., 2014; Sun R. et al., 2023; Wang et al., 2009; 2014; Zhong et al., 2018). In the current study, the structural analysis of the 42 AsSPXs revealed the presence of eight different motifs, 11 protein compositions, and intron and exon distributions across all AsSPX subfamilies (Figure 2). We found that proteins in the same subfamily exhibited similar distribution patterns, suggesting that they might be conserved and perform similar functions. On the contrary, the proteins in different subfamilies exhibited varying structures, suggesting different functions. These variations might be attributed to the evolution of the SPX proteins, resulting in a high degree of conservation and diversity. Subcellular localization prediction (Supplementary Table S2) showed that AsSPXs are mainly located on the plasma membrane, followed by cytoplasm, nucleus, and mitochondria, suggesting that these proteins exhibit varying functions. Similar distribution of SPXs has been reported in wheat (Kumar et al., 2019), soybean (Nezamivand-Chegini et al., 2021), *A. thaliana*, and rice (Duan et al., 2008; Duan et al., 2008; Wang et al., 2009). However, the SPXs of maize (Xiao et al., 2021) exhibit a varied distribution on the nucleus and chloroplast. These findings suggested that the members of the SPX protein family also exhibit varying functions across different species. Phylogenetic tree analysis (Figures 1, 2) showed that oat is evolutionarily closer to wheat, followed by barley. Similar to oat, most of the SPXs of wheat, barley, and rice belonged to the SPX group, followed by the SPX-EXS, SPX-MFS, and SPX-RING subfamilies. These results indicated that SPXs primarily function as regulators of phosphate starvation in plants (Zhang et al., 2016). However, in maize, most ZmSPXs primarily belonged to the SPX-MFS subfamily. Previous studies have shown that ZmSPXs mainly act as vacuolar Pi transporters, and cytoplasmic Pi concentration is maintained via Pi transport into vacuoles under normal phosphorus conditions (Wang et al., 2012a). The SPX-EXS subfamily proteins play an important role in phosphate acquisition, translocation, and partitioning (Wang et al., 2004; Zhao et al., 2019). The SPX-RING subfamily proteins maintain phosphate homeostasis and are involved in the Pi response (Yang et al., 2017; Yue et al., 2017). Our findings could be used as a basis for the study of the evolution and function of the SPX protein family.

### 4.2 SPXs are vital to coping with abiotic stresses

Oat is considered a highly beneficial crop. It is known to regulate blood sugar and lipid levels, exhibit anti-cancer effects, and delay aging (Paudel et al., 2021; Varma et al., 2016). Oat plants are also known for their resilience to extreme environments (Canales et al., 2021; Dangi, 2020; Oraby and Ahmad, 2012). Therefore, it is important to analyze the coping mechanisms of oat to abiotic stresses to improve the ecological environment, promote the development of agriculture and animal husbandry, and improve human health. As a phosphorus-responsive protein, SPX plays an important role under phosphorus-deficient conditions. Previous studies have shown that SPX regulates nitrogen and phosphorus balances (Ueda et al., 2020). However, only a few studies have explored the role of SPXs in enhancing cold resistance (Wang et al., 2013; Zhao et al., 2009) and salinity-alkali tolerance (Oraby and Ahmad, 2012) in plants. In the present study, we analyzed the structural composition, PPIs, and phenotypic characteristics of AsSPXs (Supplementary Figures S2, S3; Supplementary Table S8). Furthermore, qRT-PCR showed that some *AsSPXs* were also induced under cold and salt stresses, providing a better understanding of the varied roles of AsSPXs.

Phylogenetic tree analysis (Figures 1, 2) and subcellular localization prediction (Supplementary Table S2) showed that AsSPX4-2C belonged to the SPX-MFS subfamily and was located on the plasma membrane, indicating that the SPX-MFS subfamily members primarily function as vacuolar Pi transporters. Furthermore, AsSPX8-4A and AsSPX9-4C belonged to the SPX subfamily, and their genes were located in the fourth chromosome. These findings suggested that they performed similar functions, acting as regulators of phosphate starvation. Finally, AsSPX18-5D belonged to the SPX-RING subfamily, suggesting its potential involvement in the Pi reaction and phosphate homeostasis in the nucleus and extracellularly.

## 5 Conclusion

In this study, a total of 169 SPX genes were identified across five closely related oat species, distribution uneven across four subfamilies: SPX, SPX-MFS, SPX-EXS, and SPX-RING. The *AsSPXs* within each subfamily exhibited remarkable similarity in conserved motifs, gene structures, and gene duplications. Furthermore, the promoter regions of *AsSPXs* were enriched with 17 cis-acting elements associated with responses to light, low temperature, ABA, and drought. Expression analysis revealed the pivotal role of SPX genes in phosphorus sensing and responses to salt, drought, cold, and ABA stresses, underscoring the multifaceted biological functions and redundancy mechanisms within the AsSPX family. Our results provided valuable insights for future investigations on the evolutionary and functional aspects of the *AsSPX* gene family.

## Data Availability

The original contributions presented in the study are included in the article/Supplementary Material, further inquiries can be directed to the corresponding authors.
