# Supplementary figures and images for: Genome-wide identification and expression analysis of phosphate-sensing SPX proteins in oats
Source: Front Genet. 2024 Nov 20;15:1469704. doi: 10.3389/fgene.2024.1469704 (PMC11614802; doi:10.3389/fgene.2024.1469704)

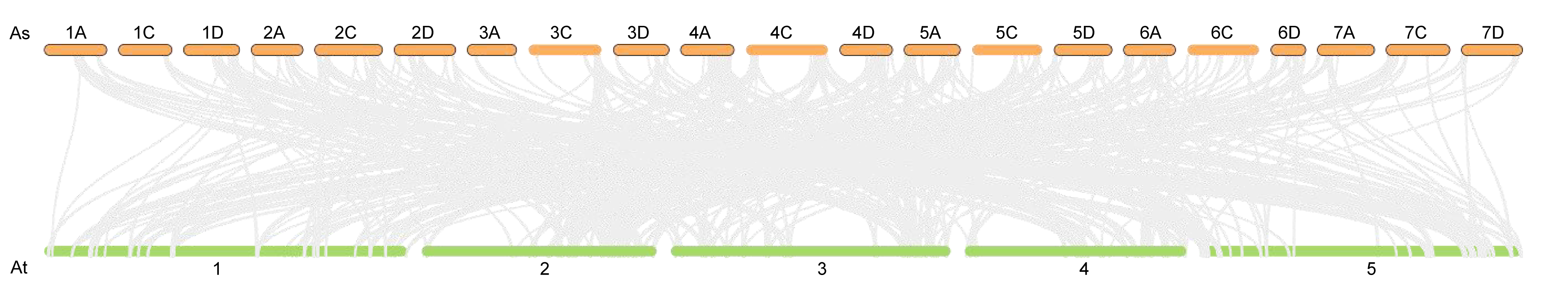

Supplement: Supplementary file 1 [file DataSheet1.zip › Supplementary files/Fig.S1.tif]

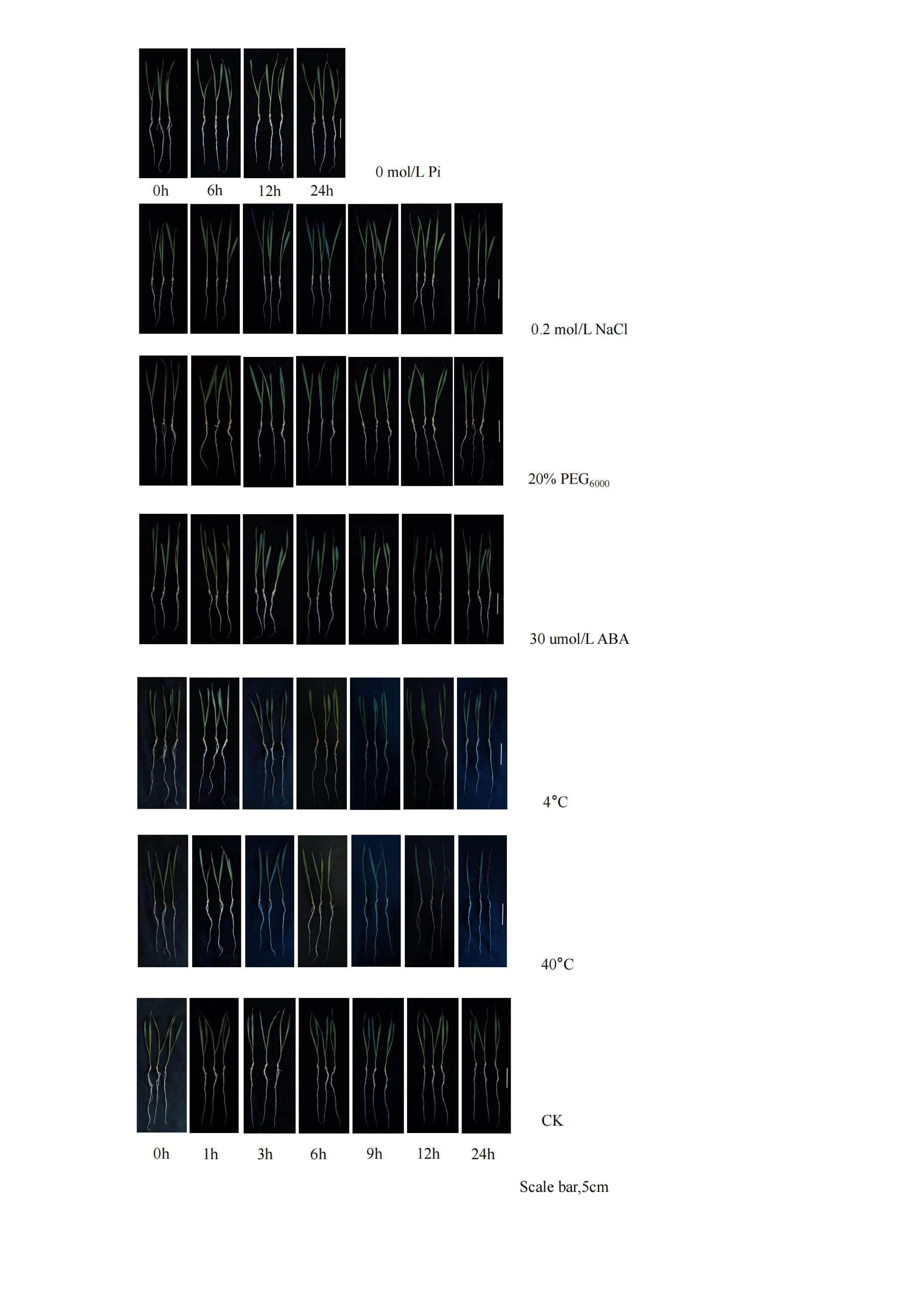

Supplement: Supplementary file 1 [file DataSheet1.zip › Supplementary files/Fig.S2 .tif]

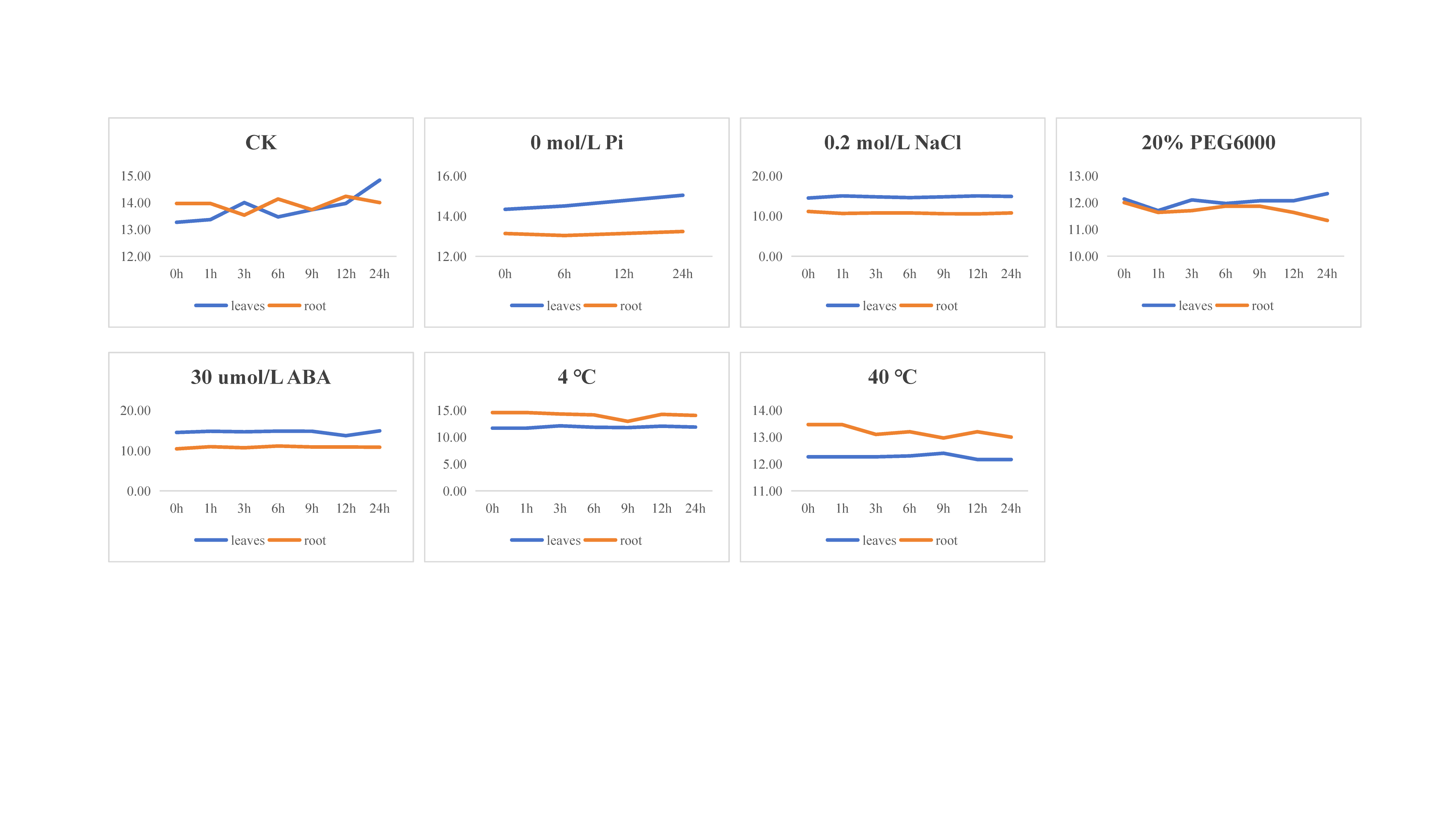

Supplement: Supplementary file 1 [file DataSheet1.zip › Supplementary files/Fig.S3.tif]
